# Divergence and diversification in North American Psoraleeae (Fabaceae) due to climate change

**DOI:** 10.1186/1741-7007-6-55

**Published:** 2008-12-17

**Authors:** Ashley N Egan, Keith A Crandall

**Affiliations:** 1 Department of Microbiology & Molecular Biology, Brigham Young University, 773 WIDB, Provo UT 84602, USA; 2 Department of Biology, Brigham Young University, Provo, UT 84602, USA; 3 Monte L Bean Life Science Museum, Brigham Young University, Provo, UT 84602, USA

## Abstract

**Background:**

Past studies in the legume family (Fabaceae) have uncovered several evolutionary trends including differential mutation and diversification rates across varying taxonomic levels. The legume tribe Psoraleeae is shown herein to exemplify these trends at the generic and species levels. This group includes a sizable diversification within North America dated at approximately 6.3 million years ago with skewed species distribution to the most recently derived genus, *Pediomelum*, suggesting a diversification rate shift. We estimate divergence dates of North American (NAm) Psoraleeae using Bayesian MCMC sampling in BEAST based on eight DNA regions (ITS, *waxy*, *matK*, *trnD-trnT*, *trnL-trnF*, *trnK*, *trnS-trnG*, and *rpoB-trnC*). We also test the hypothesis of a diversification rate shift within NAm Psoraleeae using topological and temporal methods. We investigate the impact of climate change on diversification in this group by (1) testing the hypothesis that a shift from mesic to xeric habitats acted as a key innovation and (2) investigating diversification rate shifts along geologic time, discussing the impact of Quaternary climate oscillations on diversification.

**Results:**

NAm Psoraleeae represents a recent, rapid radiation with several genera originating during the Pleistocene, 1 to 2 million years ago. A shift in diversification rate is supported by both methods with a 2.67-fold increase suggested around 2 million years ago followed by a 8.73-fold decrease 440,000 years ago. The hypothesis that a climate regime shift from mesic to xeric habitats drove increased diversification in affected taxa was not supported. Timing of the diversification rate increase supports the hypothesis that glaciation-induced climate changes during the Quaternary influenced diversification of the group. Nonrandom spatial diversification also exists, with greater species richness in the American Southwest.

**Conclusion:**

This study outlines NAm Psoraleeae as a model example of a recent, rapid radiation. Diversification rate shifts in NAm Psoraleeae are not due to current climate regimes as represented by habitat, but instead to past global climate change resulting from Quaternary glaciations. NAm Psoraleeae diversification is a good example of how earthly dynamics including global climate change and topography work together to shape biodiversity.

## Background

The legume family (Fabaceae) is the third largest plant family on earth with an estimated 19,000 species [1]. A study of diversification across angiosperms found Fabales (that is, Fabaceae, Quillajaceae, Polygalaceae, and Surianaceae) as one of 13 angiosperm orders to exhibit higher than expected diversification rates [2]. Within Fabales, Fabaceae exhibits greater diversification than the other relatively species-poor families. Furthermore, within Fabaceae, the subfamily Papilionoideae contains the bulk of species (~14,000) compared with Mimosoideae and Caesalpinioideae. This uneven distribution of species diversity continues down to lower taxonomic levels in Papilionoideae with *Astragalus *containing upwards of 2500 species [3,4]. Although *Astragalus *does not exhibit higher diversification rates relative to other related genera, the Astragalean clade, comprising *Astragalus *and a subset of the Galegeae tribe, does [3]. Other studies of Fabaceae have examined diversification rates and timing of divergence, focusing on generic or higher levels in the family [5,6]. The collection of research discussed above provides precedent for uneven diversification and shifts in diversification rates within Fabaceae, phenomena relatively unexplored at the species level (but see [7,8]).

Studies based on chloroplast coding genes *matK *and *rbcL *found elevated nucleotide substitution rates for the tribe Psoraleeae relative to other legume clades, suggestive of rapid evolution or diversification [5]. In addition, research on transcontinental clades estimated the age of the divergence between two Psoraleeae genera, *Cullen *of Australia, and *Rupertia*, endemic to the western United States, at approximately 6.3 million years ago (mya) [5]. This age estimate illustrates the recent diversification of this group, especially as the North American (NAm) clade of Psoraleeae diversified after this transcontinental split. Evidence suggests that Psoraleeae represents a recent, rapid radiation, making this group well suited to a species-level diversification study within Fabaceae.

Current circumscription of the Psoraleeae tribe includes a monophyletic New World group of six genera: *Hoita *(three spp.), *Orbexilum *(nine spp.), *Pediomelum *(29 spp.), *Psoralidium *(three spp.), and *Rupertia *(three spp.) from North America and *Otholobium *(six to eight spp.) in South America [9,10]. Four Old World genera also exist: *Cullen, Bituminaria, Psoralea*, and *Otholobium*, the latter of which spans the Old and New Worlds [10]. When considering the NAm clade, the skewed distribution of species richness towards *Pediomelum *suggests a shift in diversification rate within NAm Psoraleeae.

Diversification is the net speciation and extinction of a group. When coupled with evolutionary and ecological processes, diversification is central to the creation of species richness. Differential species richness across taxonomic and geographic levels has been widely studied, with many hypotheses proposed to explain this variation (reviewed by [11,12]). Most hypotheses concerning species richness focus on the impact of climatic factors on the diversification of species across geographic regions, endeavoring to find and explain global patterns of biodiversity. Hypotheses concerning the cause of diversification rate shifts, or net changes in speciation or extinction rate that bear on relative species diversity, have also been suggested including geographic range fragmentation, ecology, competition, key innovations, and climate change [13-18]. Climatic variables such as water and temperature account for much of the variation in species richness seen globally [19,20]. Indeed, climatic variables have been implicated in studies of species richness and diversification of a wide range of organisms, including angiosperms [21], amphibians [22], reptiles [23], vertebrates [24], insects [25], and birds [26,27], among others [19].

Hypotheses of climate change are particularly relevant to NAm Psoraleeae as climatic variables may affect habitat and range variation past and present. The five NAm genera span a variety of ecological habitats: *Hoita *is riparian, living strictly in moist soils along streams and springs in California; *Rupertia *resides on moderately moist slopes in oak-pine communities of California; *Orbexilum *resides in the semi-moist soils of forest communities of the Southeastern United States; *Psoralidium *lives in the semi-dry soils of Midwestern and Intermountain grasslands and deserts; and *Pediomelum *has adapted to well-drained soils in dry, arid ecosystems of the deserts of the Intermountain West and the grasslands and rock outcrops of the Midwestern United States [10,28,29]. Many habitats of endemic *Pediomelum *species are characterized by arid, hot environments such as the cedar glades of Tennessee, the sandy deserts of the Southwestern United States, or the limestone outcrops of the Edwards Plateau in Texas.

Observation of NAm Psoraleeae in light of evolutionary relationships suggests a shift in climate regimes with *Pediomelum*, the largest genus, and *Psoralidium *shifting to more xeric habitats, perhaps leading to increased diversification in these genera relative to other NAm Psoraleeae. This leads to the postulate that adaptation to xeric habitats may have acted as a key innovation, a trait whose origin has spurred diversification within a group [30].

The young age estimate of the tribe suggests that NAm Psoraleeae may have diversified in part in the Quaternary era, a period replete with climate oscillations brought on by glacial cycles. These shifts greatly influenced the range and distribution of species across North America, as ranges were contracted and expanded repeatedly. The onset of climate change required plant species to move, adapt, or go extinct. Upon glacial recession, new environments were open to colonization or expansion, often requiring adaptation of existing flora [31,32]. These cycles could influence speciation either through genetic differentiation spurred by range fragmentation into glacial refugia or through admixture or introgression as previously isolated entities mixed and created new and differing gene pools which contributed to speciation [33]. These events, coupled with selection pressures caused by new environments or competitors, may have spurred genetic and/or morphological change, perhaps influencing the tempo of diversification. The idea of Quaternary climate shifts impacting genetic divergence and diversification is not new [34,35]. While most studies involving the influence of this time period investigate intraspecific and population-level genetic divergence (for example,[36,37]), studies concerning the impact of this era on speciation also exist but are more rare (for example, [31,38]). If NAm Psoraleeae diversified during this time, the tempo of diversification of this group may have been influenced by these climate shifts.

In this study, we present NAm Psoraleeae as a model study system for investigating the impact of climate change on diversification within a recent, rapid radiation. We test the hypothesis of a diversification rate shift within NAm Psoraleeae against the null hypothesis of no shift in diversification rate within the group. We then explore diversification in light of various climate-related hypotheses. In particular, we test the hypothesis that a shift to xeric habitats acted as a key innovation, thus spurring a diversification rate increase in *Pediomelum *and *Psoralidium *as opposed to the rest of NAm Psoraleeae against the null hypothesis of no net increase in diversification rate of these genera. We also investigate the impact of Quaternary climate oscillations on diversification of NAm Psoraleeae. To do so, we estimate divergence times of NAm Psoraleeae to determine if the group was diversifying during this period. We then correlate diversification rate shifts to geologic time. The young age of this group provides a glimpse into the emerging evolution of NAm Psoraleeae and the impact of changing environments and climes on its radiation. In addition, the knowledge gained herein may provide insight into other species radiations that have endured similar conditions, and serves as a model for the study of diversification rates and ecological fluctuations in the robust phylogenetic context of a recent, rapid radiation.

## Results

### Divergence dates

**Figure 1:**
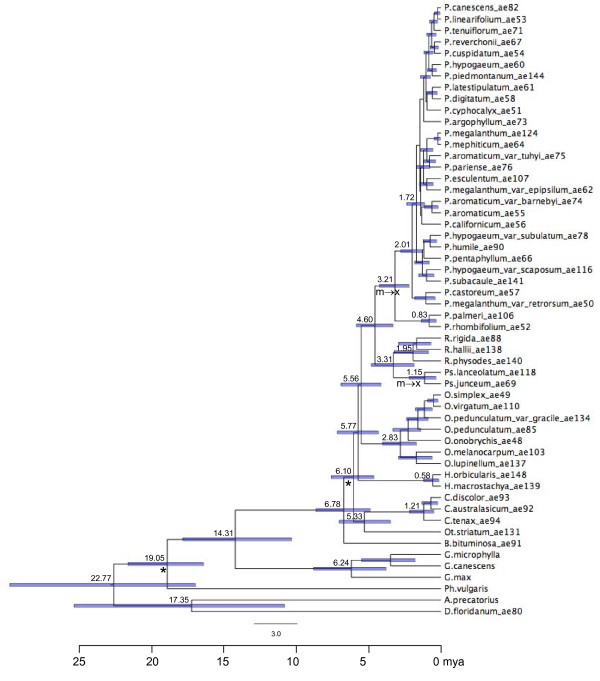
**Chronogram representing the maximum clade credibility tree estimated in BEAST based on total evidence across eight DNA regions using mixed models**. Mean divergence dates shown for key nodes. Gray bars represent the 95% high posterior density credibility interval for node age. * nodes used as calibration points. Arrow denotes shift from/to mesic (m) or xeric (x) habitat hypothesized along the lineage shown. Ingroup: P = *Pediomelum*; Ps. = *Psoralidium*; R. = *Rupertia*; O. = *Orbexilum*; H. = *Hoita*. Outgroup: C. = *Cullen*; Ot. = *Otholobium*; B. = *Bituminaria*; *G. = Glycine*; *Ph. = Phaseolus*; *D. = Desmodium*. Numbers following taxon names represent accession numbers with reference to previous work (see [9]).

A molecular clock was rejected for each data set (*p *< 0.00001; 52 degrees of freedom), justifying the use of a relaxed molecular clock for divergence dating. The mixed model combined analysis from BEAST yielded a maximum clade credibility tree very similar to previously published phylogenies [9], suggesting a monophyletic NAm Psoraleeae (Figure 1). Individual gene analyses differed somewhat in their divergence date estimates of key nodes across Psoraleeae, illustrating the variation in rates of molecular evolution and phylogenetic information content inherent in each gene region (Table 1). For the most part, date estimates determined using nuclear markers are similar to or younger than dates based on chloroplast markers. Two exceptions to this pattern exist: the node representing the most recent common ancestor (MRCA) of *Rupertia *has the minimum date (ITS) and the maximum date (*waxy*) estimated by nuclear markers, while the dates estimated for the MRCA of *Glycine *and Psoraleeae by nuclear genes exceed those estimated by chloroplast markers.

**Table 1:** Divergence dates of key nodes estimated using BEAST

MRCA of:	matK	rpoB- trnC	trnD- trnT	trnL- trnF	trnS- trnG	trnK	waxy	ITS	Ave (Genes)	Total
Glycine & Phaseolus*	18.68	18.90	18.46	18.67	18.50	18.64	18.82	18.36	18.63	19.11
SD	0.028	0.213	0.033	0.023	0.026	0.037	0.027	0.032	0.183	0.038
95% HPD lower	16.01	16.16	15.77	16.06	15.61	16.02	16.20	15.56		16.54
95% HPD upper	21.42	21.51	21.17	21.43	21.15	21.51	21.56	21.03		21.80
Cullen & Rupertia*	6.62	6.31	6.82	6.56	6.74	6.59	6.36	6.90	6.61	6.08
SD	0.024	0.019	0.039	0.024	0.026	0.033	0.030	0.032	0.206	0.055
95% HPD lower	5.10	4.72	5.37	4.91	5.07	5.06	4.69	5.45		4.61
95% HPD upper	8.17	7.89	8.45	8.14	8.44	8.29	7.97	8.45		7.56
*Glycine & Psoraleeae*	13.09	14.28	14.87	13.02	16.16	12.33	18.70	16.76	14.90	14.32
SD	0.125	0.240	0.247	0.157	0.398	0.175	0.055	0.244	2.180	0.145
95% HPD lower	8.27	8.89	9.17	7.75	7.14	7.61	15.58	9.80		10.39
95% HPD upper	18.64	20.83	20.54	19.05	24.42	17.46	21.97	20.77		17.95
*Psoraleeae*	6.67	9.55	7.10	6.89	9.13	8.12	7.08	7.12	7.71	6.77
SD	0.026	0.107	0.044	0.066	0.368	0.131	0.116	0.069	1.099	0.059
95% HPD lower	5.01	5.85	5.13	4.62	4.73	5.24	4.19	5.27		4.91
95% HPD upper	8.32	13.82	9.34	9.58	17.36	11.63	10.57	8.84		8.69
*Orbexilum*	3.67	2.94	3.82	3.54	4.04	3.88	2.66	3.03	3.45	2.83
SD	0.064	0.049	0.034	0.055	0.132	0.065	0.056	0.107	0.506	0.038
95% HPD lower	1.58	1.04	2.20	1.02	0.76	1.68	0.88	1.47		1.72
95% HPD upper	6.07	5.09	5.62	6.66	8.46	6.11	4.82	5.08		4.06
*Leucocraspedon & Psoralidium/Rupertia*	5.07	8.60	6.05	6.08	9.16	6.51	4.66	5.26	6.42	4.60
SD	0.043	0.095	0.047	0.081	0.450	0.134	0.136	0.046	1.638	0.050
95% HPD lower	3.35	5.28	4.32	3.53	3.76	3.90	2.51	3.62		3.34
95% HPD upper	6.68	12.61	7.69	8.93	18.31	9.84	7.81	7.07		5.88
*Leucocraspedon & Pediomelum*	4.02	6.46	4.52	4.79	6.49	4.61	3.62	3.56	4.76	3.21
SD	0.042	0.098	0.044	0.061	0.274	0.074	0.098	0.051	1.149	0.040
95% HPD lower	2.51	3.62	3.04	2.89	2.83	2.52	1.85	2.11		2.21
95% HPD upper	5.49	9.73	6.08	6.93	12.14	6.57	5.74	5.12		4.27
*Leucocraspedon*	1.46	1.96	1.51	1.10	1.88	1.70	1.29	0.12	1.38	0.83
SD	0.023	0.041	0.022	0.027	0.103	0.044	0.034	0.007	0.584	0.013
95% HPD lower	0.40	0.27	0.49	0.03	0.03	0.34	0.23	0.00		0.34
95% HPD upper	2.70	4.33	2.82	2.77	4.83	3.54	2.69	0.44		1.38
*Pediomelum*	3.95	3.78	2.03	2.85	6.46	2.91	2.17	2.06	3.28	2.01
SD	0.041	0.064	0.025	0.037	0.281	0.045	0.056	0.048	1.483	0.033
95% HPD lower	2.49	1.76	1.13	0.92	2.62	1.27	0.88	1.08		1.25
95% HPD upper	5.53	6.15	3.09	4.69	12.18	4.55	3.70	3.23		2.87
*Psoralidium & Rupertia*	3.91	2.15	3.41	1.23	6.92	4.44	6.23	4.47	4.09	3.31
SD	0.035	0.031	0.035	0.022	0.401	0.082	0.147	0.053	1.901	0.051
95% HPD lower	2.10	0.41	1.55	0.10	0.84	2.00	3.46	2.48		1.87
95% HPD upper	5.77	4.20	5.55	2.94	15.51	7.17	9.92	6.39		4.84
*Psoralidium*	2.76	0.41	0.55	0.33	2.41	1.22	1.87	1.50	1.38	1.15
SD	0.033	0.010	0.011	0.077	0.192	0.030	0.040	0.040	0.925	0.021
95% HPD lower	0.97	0.00	0.00	0.00	0.01	0.03	0.23	0.34		0.36
95% HPD upper	4.60	1.30	1.54	1.09	8.68	3.05	3.91	2.95		2.23
*Rupertia*	1.99	2.28	1.96	3.13	5.14	2.10	5.92	1.97	3.06	1.95
SD	0.027	0.032	0.032	0.035	0.162	0.042	0.092	0.048	1.583	0.048
95% HPD lower	0.55	0.54	0.59	0.84	0.74	0.57	3.14	0.55		0.87
95% HPD upper	3.79	4.36	3.50	5.65	10.49	4.05	8.81	3.61		3.24
*Hoita*	0.92	1.51	0.44	1.39	1.58	2.87	1.22	0.75	1.34	0.58
SD	0.018	0.026	0.010	0.028	0.062	0.120	0.035	0.032	0.735	0.011
95% HPD lower	0.02	0.07	0.01	0.02	0.05	0.08	0.03	0.05		0.15
95% HPD upper	2.42	3.59	1.25	3.55	4.13	7.24	3.56	1.80		1.21
*Cullen*	1.15	2.12	1.46	1.45	1.57	0.99	1.76	1.52	1.50	1.21
SD	0.018	0.034	0.028	0.022	0.030	0.028	0.045	0.037	0.347	0.023
95% HPD lower	0.08	0.32	0.22	0.08	0.01	0.02	0.45	0.40		0.50
95% HPD upper	2.65	4.20	3.04	3.52	4.08	2.56	3.28	3.13		2.19
*Glycine*	4.50	5.78	6.47	6.26	5.45	5.77	8.25	4.99	5.93	6.24
SD	0.090	0.143	0.135	0.106	0.167	0.107	0.272	0.136	1.132	0.115
95% HPD lower	1.11	1.31	2.50	2.30	0.67	1.85	3.89	1.82		3.82
95% HPD upper	8.43	10.84	10.75	11.05	11.54	10.02	14.07	9.31		8.87

MRCA is most recent common ancestor. SD is standard deviation. 95% upper and lower HPD is high posterior density credibility interval. Ave is the average of the dates across individual gene estimates with accompanying standard deviation below. Total is the date based on the combined dataset using mixed models.

Considering the gene regions as a whole, the combined mixed model analysis estimated the divergence date of calibrated nodes closer to the date expected than an analysis that averaged date estimates across all gene regions. For example, the calibrated node of the MRCA of *Glycine *and *Phaseolus *should have had a mean of 19.2 mya; the combined analysis estimated this divergence date at 19.1 mya while the average across the individual genes for this node was 18.6 mya (Table 1). With the exception of *Glycine *and the MRCA of *Glycine *and *Phaseolus*, the mean divergence date summarized across individual genes is older than the mean divergence date estimated under the combined mixed model analysis.

The minimum and maximum divergence date estimates across the individual genes could be taken as a range of uncertainty surrounding the divergence date of respective nodes, much the way we utilize the credibility intervals from Bayesian analyses. The ranges suggested across individual gene regions differ somewhat from the 95% high posterior density (HPD) credibility interval determined in the combined mixed model analysis. With the exception of the MRCAs of *Glycine/*Psoraleeae, *Orbexilum*, *Cullen*, and *Glycine*, the range obtained from the minimum and maximum estimates across individual gene regions is more conservative (wider) than the Bayesian credibility interval from the combined analysis (see Table 1).

While divergence estimates from the combined mixed model analysis differed from the average across individual gene estimates, the difference is slight. Furthermore, the divergence ranges suggested by utilizing the minimum and maximum estimates across individual genes or the range suggested by the 95% confidence interval (+/- 2 standard deviations) inferred using the mean across individual genes both exhibit considerable overlap with the range suggested by the 95% HPD credibility interval. We therefore confine our discussion to the dates and ranges observed under the combined mixed model framework.

As estimated in the combined analysis, Psoraleeae began diversifying in North America around 5.8 mya with the split of the genus *Hoita *from the remaining taxa (Figure 1). *Orbexilum *split from the ancestor of *Pediomelum*, *Psoralidium*, and *Rupertia *shortly thereafter, with the genus beginning to diversify around 2.8 mya (see Table 1 for credibility intervals). Around 4.6 mya, the *Pediomelum *lineage split from the lineage leading to *Psoralidium *and *Rupertia*, two genera which would later split around 3.3 mya. *Rupertia *began diversifying around 1.95 mya while *Psoralidium *speciated around 1.15 mya. *Pediomelum*, the largest genus, began diversifying around 3.2 mya.

### Differential diversification rates

The results of the seven whole-tree statistics computed in SymmeTREE using the 12 maximum parsimony tree topologies suggest a significant shift in diversification rates within NAm Psoraleeae with strong support (all statistics' *p*-value < 0.00001). The delta statistics, those that help to localize the diversification rate shift to a certain area of the phylogeny, did not find statistical support for a single shift point of diversification rate. However, the test does support that a shift took place, either gradually over the whole phylogeny or in multiple places over time.

**Figure 2:**
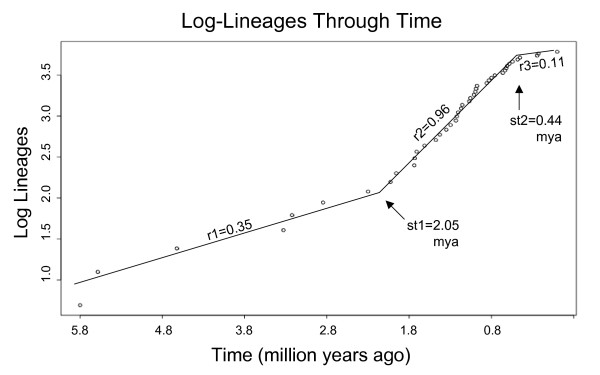
**Lineage through time plot for diversification in North American Psoraleeae based on the total evidence maximum clade credibility chronogram (see Figure **1**)**. All outgroups were culled from the chronogram until only North American Psoraleeae taxa remained, leaving *Hoita, Rupertia, Psoralidium, Orbexilum*, and *Pediomelum. st *= time of diversification rate shifts from yule3rate model estimates. *r *= diversification rate. Mya = million years ago.

The birth-death likelihood (BDL) analysis based on the maximum clade credibility tree from BEAST determined the maximum likelihood estimate of the speciation rate under the pureBirth model to be 0.58 speciation events per million years for the combined data. BDL chose pureBirth as the best rate-constant model (Table 2). It was therefore used as the null to calculate *ΔAICrc *for the rate variable models. The critical value for *ΔAICrc*, as compared with the best rate-constant model, to maintain a significance level of *α *= 0.05 is 7.96. BDL found yule3rate as the best rate-variable model (*ΔAICrc *= 11.732). We therefore reject the null hypothesis of rate-constancy because the observed *ΔAICrc *> 7.96. According to the scenario suggested by the yule3rate model, NAm Psoraleeae began diversifying with a net diversification rate of 0.35 speciation events per million years. A shift in net diversification rate took place 2.05 mya in which the rate shifts dramatically to 0.96 speciation events per million years. The net diversification rate shifted again around 0.44 mya, decreasing to 0.11 speciation events per million years (Table 2). A lineage through time plot illustrates the definitive 3-rate model as outlined in the BDL analysis (Figure 2). Combined evidence from the BDL and divergence date analyses places the diversification rate shift at the origin of the main *Pediomelum *radiation.

**Table 2:** Results of fitting diversification models to North American Psoraleeae using Birth-Death Likelihood

	pureBirth	BD	DDL	DDX	yule2rate	yule3rate
Parameters	*r *1 = 0.58	*r *1 = 0.58	*r *1 = 0.93	*r *1 = 0.74	*r *1 = 0.76	*r *1 = 0.35
		*a *= 0	*k *= 68.18	*x *= 0.081	*r *2 = 0.11	*r *2 = 0.96
					*st *= 0.45	*r *3 = 0.11
						*st *1 = 2.05
						*st *2 = 0.44
*Ln(L)*	54.723	54.723	56.242	54.829	61.654	64.588
*AIC*	-107.445	-105.445	-108.484	-105.658	-117.308	-119.177
Δ*AIC*	0	2	-1.039	1.787	9.863	11.732

The maximum clade credibility chronogram was calibrated such that the divergence of *Hoita *and *Pediomelum *was scaled to 5.8 mya. *r *= net diversification rate (speciation events per million years); *a *= extinction fraction; *st *= time of rate shift (mya); *k *= carrying capacity parameter; *x *= rate change parameter; *Ln(L) *= Log-likelihood; *AIC *= Akaike information criterion; Δ*AIC *= change in AIC relative to pureBirth.

### Testing of macroevolutionary hypotheses

The hypothesis of xeric habitats acting as a key innovation in the diversification of NAm Psoraleeae based on the 1000 posterior distribution trees from the BEAST analysis was rejected (*p*-value = 0.80) using Ree's key innovation test. The hypothesis that Quaternary climate shifts influenced diversification of NAm Psoraleeae is supported by the acceptance of the yule3rate model having a diversification rate shift, *st*, between 2.5 and 0.8 mya, the time span equating to glaciation-induced climate oscillations during the Quaternary [39]. This translates to a 2.67-fold increase in net diversification rate around 2 mya (Table 2; Figures  and 2).

## Discussion

### Recent, rapid radiations

The legume family is somewhat remarkable in that age estimates of this incredibly diverse group are relatively young, with transcontinental crown clade ages ranging from 8 to 16 mya and intracontinental crown clade ages ranging from 4 to 6 mya, providing a mostly Quaternary to Neogene age distribution [5,6]. While *Astragalus *is the legume poster child for rapid radiations, other recent, rapid radiations exist within Fabaceae [40,41]. Such radiations are often characterized by short branch lengths, little sequence variation, and diverse morphology and ecological habitats [42]. NAm Psoraleeae, especially *Pediomelum*, exhibits all of these characteristics [9,10]. This, coupled with evidence of increased rates of molecular evolution and recent age estimates of transcontinental clades, suggests that tribe Psoraleeae is a recent, rapid radiation [5].

### Divergence dates

Our divergence date estimates place the origin of NAm Psoraleeae between 4.2 and 7.5 mya with a mean estimate of 5.8 mya, concomitant with many other legume genera [6]. Of particular interest in terms of divergence dates is the largest genus, *Pediomelum*. Grimes' circumscription segregates *Pediomelum *into three subgenera based on branching pattern and dehiscence of inflorescences: subgenus *Leucocraspedon *includes *P. rhombifolium *and *P. palmeri *with remaining taxa split between subgenera *Disarticulatum *and *Pediomelum *[10]. The diversification of this genus began around 3 mya (Figure 1, Table 1) with the split of subgenus *Leucocraspedon *from the remainder of *Pediomelum*. The two species in subgenus *Leucocraspedon *differ greatly from the rest of the genus both morphologically and genetically [9,10], this evidenced in the divergence of the two main *Pediomelum *lineages. The remainder of *Pediomelum *began diversifying around 2 mya, constituting the main body of species richness of NAm Psoraleeae. To place this in perspective with other rapid radiations, the Hawaiian silverswords, the model for adaptive radiations, diverged around 5 mya [43]. Within Fabaceae, *Astragalus *was estimated to have diverged from *Oxytropis *around 12 to 16 mya with *Neo-Astragalus *radiating approximately 4.4 mya [44], while the Andean radiation of *Lupinus *is dated around 1.5 mya [7]. These dates place the diversification of *Pediomelum *on level ground with other well-known examples of recent radiations.

### Diversification rates

A rapid, family-wide diversification is strongly supported in the legume family [5]. Psoraleeae is no exception. Estimates of diversification rates vary depending on the model of diversification used in BDL on the maximum clade credibility tree from BEAST (Table 2). However, all our estimates of NAm Psoraleeae diversification are as fast or faster than rates determined for other notable rapid radiations: under the pureBirth model, a diversification rate of 0.58 for NAm Psoraleeae is similar to other rapid radiations including the Hawaiian Silverswords [43], mosses [45], squamate reptiles [46], and salamanders [47], all of which range between 0.4 and 0.8 speciation events per million years.

Skewed species richness to *Pediomelum *suggests a shift in net diversification rates within NAm Psoraleeae. Our analyses, including both topological and temporal methods, confirmed this hypothesis. Whole-tree statistics based on topology suggested a shift in diversification rate mid-depth in the phylogeny of NAm Psoraleeae. While a shift was strongly supported, the topological method was unable to decipher between the presence of multiple shifts in diversification rates along the phylogeny or a gradual change in diversification rate throughout the phylogeny as a whole. In addition, the topological method could not determine whether the shift or shifts were net increases or decreases in diversification rate. This lack of information is due to limitations of the topological method stemming from the fact that it relies solely on the order of branching events as opposed to the timing of branching events. The greater power of BDL, a temporal method, found two shifts in net diversification rate along the phylogeny. According to diversification rates suggested by the yule3rate model and BDL, NAm Psoraleeae diversification rates shifted dramatically with a net increase from 0.35 to 0.96 speciation events per million years approximately 2 mya. The increased rate is one of the fastest diversification rates published to date, rivaling those of ice plants in South Africa [48] and the Andean radiation of *Lupinus *[7]. The increase in net diversification rate did indeed occur at mid-depth in the phylogeny and represents a large increase in the diversification of the tribe within North America. The concordance of dates between the divergence date and BDL analyses confirms that the diversification rate shift took place as the main radiation of *Pediomelum *(subgenera *Disarticulatum *and *Pediomelum*), began diversifying around 2 mya (Figure 1, Table 2).

In addition to a net increase, analyses also provide evidence for a net decrease in diversification rate from 0.96 to 0.11 speciation events per million years around 440,000 years ago. This rate is far lower than the previous rates, suggesting a decrease in the amount of speciation or an increase in the amount of extinction or both. The low speciation rate seen at this recent time point could represent the speciation/population boundary. Diversification from this point forward to the present could be seen as existing on a population-level scale, as genetic differentiation between populations not sufficiently diverged to be distinguished as species. Furthermore, this decrease in diversification rate may be indirectly influenced by recent events, including those of anthropogenic origin. Indeed, we know for certain that human influence has been the cause of extinction within NAm Psoraleeae: *Orbexilum stipulatum*, once endemic to Rock Island, Kentucky, at Falls of the Ohio River, is confirmed extinct after the island was destroyed for construction of a dam; *Orbexilum macrophyllum *was collected from a single site in North Carolina and is presumed extinct [49]. Still other species are severely threatened, such as *Pediomelum pentaphyllum *and *P. humile*, due to the elimination of extant populations through urban development, leaving only one or two known populations alive. Estimates of more recent diversification shifts are especially dependent on the number of extant species. If the number of species is depleted through recent events such as human influence or through the lack of species recognition, it will more likely be reflected in diversification rate shifts taking place at more recent times, this due to the fact that we are estimating the diversification rate based on the number of species, instead of the number of clades.

### Diversification shifts – causes?

Uneven diversification is evident in both taxonomic and geographical realms, exemplified in the ideas of species richness and the latitudinal diversity gradient. A great number of hypotheses have been proposed as explanations for uneven diversification with many centering on key innovations or climate. While the pattern of the latitudinal diversity gradient, meaning the existence of greater species numbers centered around lower latitudes, is well documented, the mechanism(s) behind the phenomenon is unclear, leading to a plethora of hypotheses [11]. It is possible and even probable that no single hypothesis can explain such patterns [50]. Furthermore, these and other patterns could be the results of purely stochastic processes. However, evidence has shown that climatic factors are especially influential on the evolutionary history of many organismal groups (see below). As NAm Psoraleeae is within temporal and geographical range of Quaternary climate oscillations and exhibits a wide variety of climate-based habitats, we chose to address hypotheses involving climate change as possible effectors of diversification rate shifts.

#### Habitat shifts

Past climate change has impacted current climate regimes, creating a spectrum of climate-based habitats across the earth. Movement of ancestral species from one habitat to another may also have spurred diversification, relegating climate-based habitat as a key innovation. Climate regime shifts have been found to have impacted diversification across various organismal groups, including plants [51] and lizards [52,53]. *Hoita*, *Orbexilum*, and *Rupertia *all reside in more mesic environments characterized by warm, moist, humid climates whereas *Psoralidium *and *Pediomelum *reside in more xeric habitats characterized by hot, dry environments such as the deserts of the Intermountain and Southwest United States (see Figure 1). In testing habitat shifts from mesic to xeric habitats, our analyses suggest that past climate change affected diversification rates in NAm Psoraleeae more than shifts in present climate regimes as defined by current habitats. The shift to xeric habitats was not considered a key innovation in terms of increasing diversification rates within the group. Instead, current habitats could be seen as relicts from the effects of past climate change on its influence on the diversification of North American Psoraleeae.

#### Quaternary climate oscillations

The recent origin of the main radiation of *Pediomelum *around 2 mya places its diversification in the middle of the Pleistocene epoch of the Quaternary era, a time period replete with dramatic glaciation-induced climate oscillations. These glacial fluxes include Milankovitch cycles which consist of repeated formation and recession of glacial ice sheets having periodicities of 20,000 to 100,000 years, resulting in wide-ranging climate fluxes [33]. It has been well documented that much of the Earth's biodiversity and many species' evolutionary trajectories were drastically influenced by the climate fluxes brought on by Quaternary glaciations (for example, [36,37,39,54]). In North America, the sheer presence of ice sheets shifted climates and strongly influenced the range and distribution of species [55]. Ice formation may have influenced speciation via genetic differentiation through range fragmentations such as glacial refugia. Upon glacial recession, new environments were formed and subsequently colonized through species expansion and adaptation [31,32]. Given the geographical and temporal distribution of NAm Psoraleeae, these cycles may have influenced its evolutionary history. Indeed, the shift point for the increase in diversification along the phylogeny at 2 mya and the lineage through time plot from our analyses support the hypothesis that Quaternary climate fluxes, occurring between 2.5 and 0.8 mya, influenced the diversification of NAm Psoraleeae.

#### Geography

The effects of these oscillations are not homogenous, however. The Quaternary ice ages and climate oscillations affected geographic regions across North America differently. The Southeast provided several refugia in which species could survive [31] while the montane regions of the West were characterized by more varied environments such as ice, tundra, pluvial lakes, and deserts [56]. In addition, elevation played an important role in species distributions as elevational ranges of plants were 600 to 1200+ meters below modern ranges, an effect felt more readily in the topographically diverse regions of the West [57]. Upon glacial recession, the Intermountain West offered a greater variety of habitats for colonization and adaptation as a result of topography. This is in line with Cracraft's lithospheric complexity hypothesis, stating that geographic separation spurred by topographical changes promotes speciation [58]. In addition to orogeny, glacial cycles contributed to topographical change, and thus are linked with the lithospheric hypothesis.

Geographical trends are seen in the diversity of Psoraleeae. NAm Psoraleeae ranges throughout the United States: *Orbexilum *is mostly confined to the southeast United States, with one species reaching into Mexico, *Pediomelum *is mostly found in central and Intermountain states, *Hoita *is endemic to California, *Rupertia *ranges along the West Coast, and *Psoralidium *is spread over the great plains with one species endemic to Utah. Of interest is the seeming correlation of lineage age with southerly distribution. *Orbexilum *is one of the oldest NAm Psoraleeae genera, diversifying earlier than most other lineages. This could be demonstrative of long-established ranges of *Orbexilum *through Pleistocene refugia in the Southeast United States. Of import also is the age of *Pediomelum *as a whole, beginning its diversification around 3 mya with the break off of subgenus *Leucocraspedon*. The two *Leucocraspedon *species exhibit the most southern distribution of all *Pediomelum *species with *P. rhombifolium *being distributed throughout Texas and into Mexico while *P. palmeri *is almost exclusively found in Mesoamerica. This southern distribution perhaps allowed this lineage to persist through the Quaternary climate oscillations as the effects of glacial climate change were moderated toward the equator. The main radiation of *Pediomelum *exhibits the greatest number of species with the lowest intraspecific variation, findings in accord with the lithospheric complexity hypothesis, geographic separation, ecological diversification due to habitat diversity, and range fragmentation brought on by Quaternary climate shifts. The pattern of less diversity in the Southeast (as evidenced by *Orbexilum*) and greater diversity in the Southwest (as evidenced by *Pediomelum*) is also found in *Cicindela *beetles [59] and *Agelenopsis *spiders [60], among others.

## Conclusion

NAm Psoraleeae has proved useful for studying evolutionary divergence and the impact of climate change on diversification within a recent, rapid radiation. This work effectively demonstrates differential rates of diversification at the species and generic levels, trends manifested across taxonomic levels within Fabaceae. An estimation of divergence dates within Psoraleeae found that the main radiation of *Pediomelum*, comprising the bulk of species richness in NAm Psoraleeae, originated 1 to 2 mya during the Quaternary era, concurrent with the Milankovitch climate oscillations brought on by glacial cycles. The hypothesis of a diversification rate shift in NAm Psoraleeae was confirmed by both topological and temporal methods. We demonstrated a large increase in diversification rate near the beginning of the Pleistocene epoch. Our findings suggest that the diversification rate increase in NAm Psoraleeae is not due to current climate regimes as represented by habitat, but rather imply that Quaternary climate oscillations impacted the diversification of the group. We show nonrandom spatial diversification as well, with higher species diversity in the American Southwest.

NAm Psoraleeae diversification is a good example of how earthly dynamics including global climate change and topography work together to shape biodiversity. In a time when the issue of global climate change pervades society and science, this study aptly illustrates the control that global climate change can have over the evolutionary trajectories and fates of lineages and species.

## Methods

### Taxon sampling and DNA sequences

This study includes a thorough sampling of NAm Psoraleeae, with only four of 47 recognized NAm Psoraleeae taxa unsampled. Of these, *Hoita strobilina *is critically endangered, *Orbexilum macrophyllum *and *O. stipulatum *are presumed extinct, and *Pediomelum latestipulatum *var. *latestipulatum *was not collected. In addition to North American taxa, representatives of *Otholobium*, *Cullen*, and *Bituminaria *are included for estimating the dates of divergence within the tribe. Six outgroup taxa are incorporated for calibration: *Abrus precatorius, Phaseolus vulgaris, Desmodium floridanum, Glycine microphylla, G. canescens*, and *G. max cv. Essex*. In total, 54 taxa are included as described in Egan and Crandall [9].

All phylogenetic analyses were based on eight DNA regions: the Internal Transcribed Spacer (ITS) and a single-copy gene, Granule Bound Starch Synthase I (*Waxy*) from the nuclear genome and the *trnL-trnF*, *trnD-trnT*, *trnS-trnG *and *rpoB-trnC *intergenic spacers, the *trnK *intron regions, and the *matK *protein-coding gene from the chloroplast genome. DNA isolations, primer specifications, PCR amplification, DNA sequencing, voucher specimen information, GenBank numbers, and maximum parsimony phylogenetic analyses are described in Egan and Crandall [9].

### Divergence dates

Departure from a molecular clock was determined using likelihood ratio tests with scores estimated using rate-constant and rate-variable models [61] in PAUP* 4.0 [62]. Models were estimated as described in Egan and Crandall [9] using ModelTest 3.7 [63]; *matK*, *trnK*, *trnD-trnT*, *rpoB-trnC*, ITS, and the combined data set used the general time reversible (GTR) model plus proportion of invariable sites (I) and the gamma distribution (G) to account for rate heterogeneity, *trnL-trnF *used GTR+I, *trnS-trnG *used GTR+G, and *waxy *used HKY+G.

Divergence dates were estimated for individual genes and a combined total evidence dataset via Bayesian MCMC sampling conducted in BEAST v1.4.7 [64] under a relaxed clock model with log-normally distributed, uncorrelated rates of substitution between branches [65]. No topological constraints were employed, allowing topological uncertainty to be taken into account. We analyzed each of the eight DNA regions individually and the combined total evidence dataset, allowing the investigation of how rates of molecular evolution vary among DNA regions.

Two calibration points were used for estimating divergence dates in BEAST: the most recent common ancestor of *Glycine *and *Phaseolus *has a mean estimate of 19.2 mya accompanied by a standard deviation (SD) of 1.4 mya. The minimum and maximum age estimates for this node are 15.5 to 22.4 mya. The most recent common ancestor of *Cullen *and *Rupertia *was estimated to range between 4.1 to 8.8 mya with a mean date of 6.3 mya (0.9 SD). These dates were estimated from a family-wide divergence date analysis using penalized likelihood in r8s using Bayesian-inferred phylogenies for *matK *and based on 13 fossil calibration points [5]. We chose to use their *matK *date estimates over *rbcL *because the *matK *dataset incorporated more fossil calibration points (13 as opposed to 9) and the estimate of dates was more stable in the *matK *dataset as shown by a lower SD. We incorporated these dates as calibration points into our BEAST analyses by the use of a normal prior using their respective mean and SD estimates. This strategy allows uncertainty surrounding these dates as calibration points to be accounted for in our divergence date analyses.

Each gene or data set used the model of evolution determined by ModelTest mentioned above. The combined dataset incorporated mixed models into the analysis, allowing each gene to evolve under the model and parameters best suited to the gene region. The tree prior was modeled under the Yule speciation process. With the exception of the normally-distributed priors on calibration points, all other priors were set as default values in the program BEAUti v1.4.7 which was used to help create the XML files for input to BEAST [64]. Final analyses consisted of two separate MCMC runs of 5 to 10 million generations sampled every 1000 generations for the individual gene analyses, while the combined dataset was run for 15 million generations. Tracer v1.4 [66] was used to confirm likelihood stationary and adequate mixing of the MCMC chains and whether the two separate runs had converged to the same results. If runs had converged, results of both runs were combined using LogCombiner v1.4.7 [64]. Tracer v1.4 was then used to determine adequate burn-in (10%), confirm mixing and stationarity of the combined runs, and summarize the posterior distribution for divergence date estimates across key nodes. The maximum clade credibility tree was computed for the combined dataset using TreeAnnotator v.1.4.7, part of the BEAST software package.

### Differential diversification rates

Two methodological strategies exist for estimating differential diversification rates across phylogenies: comparisons of topological species distribution to a random distribution of diversity; and comparisons of temporal distribution of divergence events to a randomly created distribution [67]. Topological methods are generally employed as a first test to determine if diversification rate shifts exist across lineages, while temporal methods are more powerful at addressing specific questions of when, how much, and where shifts in diversification took place along a phylogeny. Here, we employ both strategies to test both general and specific hypotheses concerning diversification rates.

The topological program SymmeTREE [68] was used to test the general hypothesis of variation in diversification rates across NAm Psoraleeae. This tests for deviations of observed topological distribution of species diversity to that expected under the Yule model [69] of an equal-rates Markov random-branching process. Seven whole-tree tests of differential diversification rates, each sensitive to different scenarios and nodal depths, are employed. *M*_*R *_tests for diversification at deeper nodes, or at the root of the tree, by determining if the observed trees are more or less asymmetric than expected [70]. *I*_*C *_is similar but is a calculated measure instead of a probability [70]. *M*_Π _and *M*_Σ _consider the asymmetry of internal nodes, with M_Π _more sensitive to deeper nodes through multiplication of individual node probabilities and M_Σ _more sensitive to nodes closer to the tips of the tree through addition of node probabilities. *M*_Π_* and *M*_Σ_* vary from the above by having each node probability weighted by its size [71]. Unlike the above tests, *B*_1 _measures tree balance as opposed to imbalance. This statistic is sensitive to changes at the tips of the tree as it excludes the root [72]. As a topological method, SymmeTREE does not make use of branch length data, but uses order of branching events only. For the SymmeTREE analysis, we therefore used the previously estimated set of 12 fully resolved, most parsimonious trees from the total evidence maximum parsimony analysis that included gaps [9]. This allowed us the benefit of completely resolved trees and incorporation of a measure of phylogenetic uncertainty while avoiding the duplication of exact topologies that may be evident in the set of trees from the Bayesian posterior distribution. Bonferroni corrections were used to adjust significance thresholds across multiple tests.

The temporal method BDL was used to test specific hypotheses of diversification rate shifts [39,53]. This method calculates maximum likelihood estimates of speciation rate parameters and a likelihood score per tree. The null model of rate-constancy is tested against rate-variable models using the Akaike Information Criterion [73]. BDL has been found to perform as well or better than the popular γ-statistic, which only identifies temporal decreases in diversification [74], and has greater power to detect shifts in diversification rates in the presence of extinction [53]. By comparing the sum of the likelihood of internode distances across the tree under varying models, BDL can decipher between increased diversification rate vs. rate-constancy with background extinction [53]. Several diversification models were tested using LASER [75]. Rate-constant models included pureBirth, a constant speciation model (Yule) with zero extinction [69] and birth-death (BD), a constant speciation-constant extinction model [76]. Rate-variable models include density-dependent speciation models with exponential (DDX) and logistic (DDL) variants [77,78], yule2rate and yule3rate models. The yule2rate and yule3rate models are multi-rate variants of the Yule model, allowing two and three shifts in speciation rates respectively at times *st *across a tree. Branching times only were allowed as possible rate shift points. We used the maximum clade credibility tree from the combined dataset estimated in BEAST (chronogram shown in Figure 1) to test for diversification shifts using BDL. This chronogram was chosen in part due to the need for complete resolution and also to make use of divergence date estimates based on the full dataset. Outgroups were excised and the root, the ancestor of *Hoita *and *Pediomelum*, scaled to 5.8 mya – the divergence date estimated in BEAST based on the combined dataset (see Figure 1). Significance of the change in Akaike Information Criterion (*AIC*) scores was determined by creating a distribution of *AIC *scores. This was done by simulating 1000 trees using yuleSim in LASER having the same number of taxa and the same speciation rate as that estimated under the pureBirth model.

### Testing of macroevolutionary hypotheses

The factors that we tested as influencing the tempo of diversification in NAm Psoraleeae include Quaternary climate changes and shifts in climate regime from moist, warm climes (mesic) to dry, hot climes (xeric). The adaptation to or colonization of xeric environments was hypothesized as a key innovation, a trait or character change whose origin may have spurred diversification. *Pediomelum *and *Psoralidium *live in xeric environments while other NAm Psoraleeae are in more mesic habitats. Xeric environment was tested to see if its origin was associated with increased diversification in NAm Psoraleeae against the null hypothesis of no increase in diversification rates across the tribe. We used the key innovation test [79] which employs Bayesian techniques to assess correlations between the timing of branching events (representative of speciation) and the evolutionary history of the character of interest. If acting as a key innovation, lineages with the new regime will have shorter waiting times before branching, as represented by internode length, than those without it. Tree length is calibrated to equal the total amount of change in the character over the whole phylogeny, such that waiting times represent the relative time spent in one character state as opposed to the other. Significance is determined by comparison to a null distribution of random waiting times between branching events. This goes beyond clade size methods by incorporating the time of branching events and allowing the trait to be both acquired and lost. Other tests assume acquisition only, not allowing loss of the trait, thereby excluding potential information from the analysis [79]. We used a set comprising the last 1000 trees from the Bayesian (BEAST) posterior distribution based on the combined dataset to test the hypothesis of xeric environment acting as a key innovation. This treeset was optimal for this analysis because the trees were already ultrametric and allowed incorporation of phylogenetic uncertainty. Trees were scaled so that the total tree length was equal to total character change from mesic to xeric habitats. In this case the tree length was 2, illustrating two changes from mesic to xeric environment at the base of the lineages leading to *Psoralidium *and *Pediomelum *(see Figure 1).

The impacts of Quaternary climate shifts were tested using lineage-through-time plots [39] and BDL using LASER. These used the maximum clade credibility chronogram from the combined dataset estimated in BEAST. A shift in diversification rates taken between 2.5 and 0.8 mya, the time of Quaternary climate oscillations, is taken as support for their influence on diversification in this group.

## Authors' contributions

ANE gathered data, ran analyses, and wrote the paper. KAC suggested programs for analyses, gave feedback and guidance on hypotheses, and provided comments on the manuscript. Both authors read and approved the final manuscript.
